# Application of Mitral Annular Systolic Displacements and Velocities for the Evaluation of Left Ventricular Systolic Function and Reserve

**DOI:** 10.4021/cr7w

**Published:** 2011-01-20

**Authors:** Dawod Sharif, Amal Sharif-Rasslan, Camilia Shahla, Uri Rosenschein

**Affiliations:** a Department of Cardiology, Bnai Zion Medical Center, Israel; b Technion – Israel Institute of Technology, Haifa, Israel

**Keywords:** Systolic function, Dobutamine stress echocardiography, Tissue imaging, Functional reserve

## Abstract

**Background:**

Mitral annular systolic displacement from M-mode echocardiography and velocity from tissue Doppler imaging reflect subendocardial longitudinal systolic LV performance and may precede radial abnormalities. The aim of this study is to evaluate the utility of mitral annular systolic displacement (D) and velocity (V) during dobutamine stress echocardiography (DSE) in detecting left ventricular (LV) functional reserve and wall motion abnormality (WMA).

**Methods and Results:**

Fifty-nine subjects, 15 with resting WMA, underwent DSE and measurement of mitral systolic (D) and (V) before and immediately after DSE. Annular septal (D) was lower in those with WMA than in those without, at rest 10.5 ± 4 cm versus 13.2 ± 2 cm, *p* = 0.015, and after DSE, 11.7 ± 3.8 cm versus 14 ± 2.25 cm, *p* = 0.036, but without significant change after stress. Annular systolic (V) at rest with WMA was 9.7 ± 1.8 cm/sec and similar to those without, 11.25 ± 2.7 cm/sec. In both groups the velocity increased after DSE, 14.5 ± 4.5 cm/sec, *p* = 0.025 with WMA and 17.8 ± 3.2 cm/sec, *p* = 1.99 x 10^-10^ in those without WMA. Velocities after DSE were lower in those with WMA, *p* = 2.25 x 10^-6^.

**Conclusions:**

To evaluate LV systolic performance mitral annular systolic longitudinal displacement is valuable at rest, but for assessment of LV functional reserve after stress velocities are better.

## Introduction

Left ventricular myocardial fibers are arranged in concentric and oblique fashions in a spiral course, as depicted already in 1669 by Richard Lowers "tractatus de corde" [1]. Assessment of left ventricular systolic function is fundamental for adequate diagnosis and management of cardiac patients with overt and covert heart failure. Usually radial left ventricular systolic performance resulting from shortening of the concentric fibers is evaluated. However longitudinal abnormalities reflecting subendocardial myocardial longitudinal fiber function may precede radial-circumferential abnormalities in healthy individuals [2]. In addition, it was reported that in the presence of abnormal long axis function, stroke volume was reduced despite that the ejection fraction was preserved [3].

Standard M-mode echocardiography of the mitral annulus may be considered a reliable method for the assessment of left ventricular longitudinal function [4]. Tissue Doppler systolic velocities of the mitral annulus are considered a marker of left ventricular contractile reserve in the absence of regional dysfunction [5]. Symptoms related to left ventricular dysfunction may appear only on exercise, therefore evaluation for occult myocardial systolic dysfunction is important. The aim of the present study was to evaluate the utility of mitral annular systolic displacement (D) and velocity (V) during dobutamine stress echocardiography in the assessment of systolic LV functional reserve.

## Methods

### Population

Fifty-nine patients, 29 women, age 60.3 ± 13.4 years, were prospectively evaluated for the presence of coronary artery disease. Fifteen of the subjects had wall motion abnormality at rest. All underwent dobutamine stress echocardiography studies using the usual protocol, and measurement of mitral systolic displacement from M-mode echocardiography of the mitral annulus from apical views (D) and tissue Doppler imaging systolic velocities (V) of loci at the mitral annulus from apical views, before and immediately after the study.

### Dobutamine stress echocardiography

The protocol of dobutamine infusion consisted of 3-minute stages for each dose, staring with 5 ug/kg/min and increasing to 10, 20, 30 and 40 ug/kg/min. If end-points did not occur or 85% of the age-adjusted heart rate was not achieved, 0.25 mg atropine was injected every 2 minutes up to 1 mg or until the target heart rate was achieved. Blood pressure and 12 lead electrocardiograms were recorded at rest and throughout the dobutamine stress echocardiography study. Horizontal or downsloping > 1mm ST-segment depression at 0.06 sec after the J point was considered as evidence for myocardial ischemia.

### Image acquisition

Images were obtained while the patients in the left lateral decubitus position. A standard commercial Siemens, Acuson Sequoia echocardiography system, California, equipped with 3.5 MHZ transducers was used. All patients had complete Doppler echocardiographic studies before dobutamine stress echocardiography. Parasternal long axis and short axis as well as apical 4-chamber and 2-chamber views were recorded at rest, low dose dobutamine infusion, peak exercise and in the recovery period. Digital images were stored on magneto-optic discs for later off-line analysis. In addition super VHS videotape recordings were performed throughout the studies.

### Dobutamine stress echocardiographic analysis

Segmental left ventricular wall motion analysis was performed using 16-segment model [6]. Regional wall motion and segmental score were estimated as normal = 1, hypokinetic = 2, akinetic = 3 or dyskinetic = 4. New or worsening segmental wall motion was considered as ischemic response. Ischemic response (I) was identified when wall motion decreased by at least 1 grade in 2 adjacent segments or wall motion decreased by at least 2 grades in 1 segment, otherwise no ischemia, or normal response (N) was diagnosed. Wall motion score index (WMSI) was calculated as: WMSI = (Sum of scores of 16 segments)/16.

### Mitral annular displacement

Measurements of translations of the mitral annulus were performed from M-mode tracings from the apical views from the septal, lateral, anterior, inferior and posterior portions of the annulus.

### Tissue Doppler imaging

The apical 4 chamber, 2 chamber and 3 chamber views were used to assess the longitudinal velocities of the mitral annulus. The sample volume of the pulsed wave Doppler was located at the mitral annulus and recording was performed from the septal, lateral, anterior, inferior and posterior portions of the annulus. In addition Doppler sampling from the proximal anteroseptal segment was performed. Annular Doppler velocities were recorded on videotape for off-line analysis.

### Statistical analysis

Mean values and standard deviation of the 6 annular mitral longitudinal displacements and velocities were calculated for all. Student’s t-test assuming unequal variances was performed; *p* < 0.05 was considered significant.

## Results

All subjects underwent DSE studies safely and uneventfully. Heart rate increased from 62.4 ± 9.9 bpm to 133 ± 14.4 bpm. Systolic blood pressure increased from 138 ± 7 mmHg to 162 ± 9 mmHg.

### Left ventricular wall motion

In 26 subjects WMSI was 1 at rest and did not change after DSE, while in 18 subjects WMSI was 1 at rest and increased to 1.12 ± 0.14 after stress. In 15 subjects WMSI at rest was 1.36 ± 0.34 and increased to 1.47 ± 0.32 after DSE.

### Mitral annular displacement

Mitral annular systolic displacement did not increase after dobutamine in the presence or absence of left ventricular wall motion abnormality at rest (Table 1) or when wall motion abnormality appeared only after DSE (Table 2). However, both at rest and after dobutamine mitral systolic displacements were less in subjects with resting left ventricular wall motion abnormalities (Table 1). However, these differences in annular displacements were smaller when subjects with DSE-induced wall motion abnormality were considered separately (Table 2).

**Table 1:** Longitudinal Mitral Annular Translation (mm)

		Septal	Lateral	Inferior	Anterior	Posterior
WMA	At rest	10.5 ± 4	13.4 ± 3	12.2 ± 0.5	12.7 ± 2.6	12.3 ± 2.4
	Post-DSE	11.7 ± 3.8	14 ± 1.75	12.3 ± 1.89	11.9 ± 2.5	13 ± 0.8
	P-value*	n.s.	n.s.	n.s.	n.s.	n.s.
No WMA	At rest	13.2 ± 2	15.6 ± 2.6	15 ± 3	15.2 ± 2.6	14.5 ± 2.9
	Post-DSE	14 ± 2.25	16.4 ± 2.6	15.75 ± 2.8	15.6 ± 2.8	15.7 ± 2.3
	P-value*	n.s.	n.s.	n.s.	n.s.	n.s.
P-value‡	At rest	0.015	0.096	0.002	0.02	0.03
	Post-DSE	0.036	0.067	0.001	0.042	0.006

WMA: Wall motion abnormality

* P-value Pre and Post DSE

‡P-value with and without WMA

**Table 2:** Mitral Annular Translation and Velocities at Rest and After DSE in Different Wall Motion Subgroups

		Septal			Lateral		
		rest	DSE	p	rest	DSE	p
Translation (mm)	N	13.2 ± 1.87	14.03 ± 1.98	0.167	15.6 ± 2.64	16.4 ± 2.37	0.292
	I	13.1 ± 2.3	14.1 ± 2.5	0.28	15.4 ± 1.83	17.4 ± 3.8	0.085
	A	10.45 ± 3.04	11.66 ± 3.7	0.34	13.3 ± 3.9	13.6 ± 3.86	0.89
p - (A : N)		0.0147	0.071		0.096	0.07	
p - (A : I)		0.925	0.08		0.119	0.03	
p - (N : I)		0.885	0.94		0.81	0.365	
Velocity (cm/sec)	N	11.25 ± 3	17.8 ± 3.3	2 × 10^-10^	12.7 ± 3	17 ± 4.1	0.00012
	I	11.2 ± 4.1	18 ± 5.2	0.00035	11.1 ± 2.8	18.7 ± 5.7	0.00023
	A	9.65 ± 2.6	14.5 ± 4.9	0.025	9.6 ± 2.6	13.7 ± 4.4	0.012
p - (A : N)		0.08	0.0395		0.0049	0.037	
p - (A : I)		0.23	0.078		0.156	0.018	
p - (N : I)		0.92	0.91		0.126	0.319	

N: Normal wall motion at rest and after DSE

I: Ischemic - normal wall motion at rest and abnormal after DSE

A: Abnormal wall motion at rest and after DSE

### Mitral annular systolic velocities

Mitral annular systolic velocities increased significantly after dobutamine in those with and those without resting left ventricular wall motion abnormalities (Table 3) and in those with DSE-induced wall motion abnormality (Table 2). At rest, small and non-consistent differences were found between subjects with wall motion abnormality and those without (Table 3). However, after dobutamine a larger and more significant increase in mitral annular velocities was found in subjects without wall motion abnormality (Table 3). Subjects with DSE-induced wall motion abnormality had normal mitral annular velocities (Table 2).

**Table 3:** Mitral Annular Peak Systolic Velocity (cm/sec)

		Septal	Lateral	Inferior	Anterior	Posterior
WMA	At rest	9.7 ± 1.8	9.59 ± 2.6	9.2 ± 2.3	9.5 ± 1.4	9.3 ± 1.5
	Post-DSE	14.5 ± 4.5	13.7 ± 4.2	14.7 ± 5	18.7 ± 3.6	13.8 ± 4.8
	P-value*	0.025	4.3E-8	1.6E-8	4.5E-6	4.2E-12
No WMA	At rest	11.25 ± 2.7	12.7 ± 3.7	11.3 ± 1.8	11 ± 2.4	11.4 ± 2.4
	Post-DSE	17.8 ± 3.2	17 ± 3.7	16.9 ± 3.6	18.3 ±4.4	17.3 ± 4
	P-value*	1.99E-10	4.9E-8	1.9E-8	1.9E-5	6E-10
P-value‡	At rest	0.385	0.072	0.034	0.13	0.009
	Post-DSE	2.25E-6	0.05	0.038	0.23	0.05

WMA: Wall motion abnormality

* P-value Pre and Post DSE

‡ P-value with and without WMA

### Left ventricular longitudinal systolic function and wall motion score index

**Figure 1:**
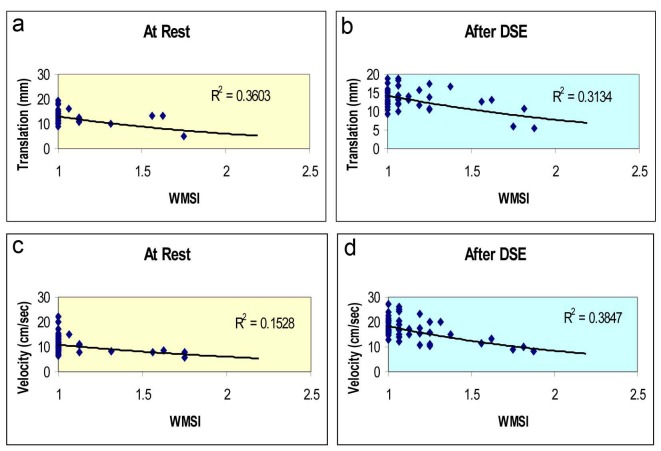
Relation between left ventricular WMSI and mitral annular translation distance at rest (a) and after dobutamine stress (b); and between mitral annular systolic velocities at rest (c) and after dobutamine stress (d).

An exponential relationship was found between systolic mitral annular displacement and left ventricular wall motion score index at rest (Fig. 1a), and after dobutamine (Fig. 1b), however with lower correlation after stress. The exponential relationship between mitral annular velocities and left ventricular wall motion score index at rest was poor (Fig. 1c), however the correlation improved after DSE and in fact was the best relationship between mitral annular parameter and LV-WMSI (Fig. 1d).

## Discussion

In this study dobutamine was used as an inotropic agent to evaluate left ventricular systolic function and contractile reserve. Left ventricular longitudinal systolic function was evaluated from mitral annular systolic displacement and from tissue Doppler mitral annular systolic velocities. Mitral annular systolic longitudinal displacement was valuable in the assessment of left ventricular systolic performance at rest and did not change significantly by dobutamine infusion, while mitral annular tissue Doppler systolic velocity increased after dobutamine infusion and differentiated between patients with and without left ventricular systolic dysfunction only after the infusion of the inotropic agent.

Left ventricular systolic longitudinal dysfunction may precede circumferential dysfunction and may be reduced in the presence of normal ejection fraction [2, 3]. Ventricular long axis function as measured from M-mode of the atrioventricular valve annuli is of major importance for long term survival in patients with heart failure [7]. Moreover, abnormalities in left ventricular longitudinal functional reserve were reported in patients with diabetes mellitus compared to presumably non-diabetic control subjects [6]. It was suggested that maximal longitudinal contraction velocity is a more sensitive index than left ventricular ejection fraction [8].

In normal subjects, the segmental response to increase in dobuatmine infusion is a gradual and continuous increase in myocardial tissue Doppler velocities, strain rate and strain [9-12]. Abnormal increase in segmental tissue Doppler velocity during stress indicates ischemia, appearance of left ventricular wall motion abnormality and apparently reduced contractile reserve [13, 14].

Dobutamine infusion caused an increase in the longitudinal projection of velocity vector more in subjects without wall motion abnormality, however the integral upon time giving the M-mode systolic displacement of the mitral annulus was similar in those with and without wall motion abnormality. Thus, in our study tissue Doppler mitral annular systolic velocities reflected contractile reserve better than M-mode mitral annular systolic displacement as was suggested in previous studies [5].

Mitral annular systolic displacement from M-mode echocardiography (D) is considered an indicator of left ventricular longitudinal systolic function [4] and in this study it did not change during DSE. Since D is the integral of the longitudinal velocity of contraction throughout the period of systole, $\left(D=\int_{0}^{\tau }\nu (t)dt\right)$, and D is not changing during DSE, and since it is known that during stress the cardiac cycle is decreasingon the account of decrease in diastole, it is concluded that the important change is in longitudinal acceleration leading to the change in velocity after DSE. Instantaneous and not mean acceleration should be evaluated which could not be measured by the available Doppler-echocardiographic equipment.

Left ventricular global systolic function was estimated in terms of wall motion score index. Mitral annular systolic tissue Doppler velocity correlated better than annular M-mode systolic displacement with wall motion score index. The correlation improved during dobutamine infusion implying that longitudinal systolic performance, especially velocities, is better exploited as a reserve for global systolic function during stress. Subjects with left ventricular wall motion abnormality appearing only after DSE were similar to normal due to small change in wall motion score index.

Thus, assessments of left ventricular systolic function are better appreciated during stress, combining both radial and longitudinal systolic performances. This finding may be used in the evaluation of timing of surgery in asymptomatic subjects with severe valvular regurgitation.

### Limitations

Exercise stress for the evaluation of left ventricular systolic function reserve may be more physiologic than pharmacologic stress, however dobutamine is widely applied agent, especially in subjects with limited exercise abilities. Measurement of acceleration of the motion of the mitral annulus may add new insight, but acceleration preferably should be simultaneous and not average values, which is not available in usual echocardiographic machines.

### Conclusions

Mitral annular systolic displacements reflect abnormalities in left ventricular systolic function and wall motion at rest but do not change during dobutamine infusion, while mitral annular velocities consistently distinguish subjects with systolic dysfunction and wall motion abnormality only after dobutamine stress. Mitral annular systolic displacements correlate with global left ventricular systolic function at rest while velocities correlate better after stress.
